# Multiplexed automated digital quantification of fusion transcripts: comparative study with fluorescent in-situ hybridization (FISH) technique in acute leukemia patients

**DOI:** 10.1186/s13000-016-0541-z

**Published:** 2016-09-15

**Authors:** Ariz Akhter, Muhammad Kashif Mughal, Ghaleb Elyamany, Gary Sinclair, Raja Zahratul Azma, Noraidah Masir, Salwati Shuib, Fariborz Rashid-Kolvear, Meer-Taher Shabani-Rad, Douglas Allan Stewart, Adnan Mansoor

**Affiliations:** 1 Department of Pathology and Laboratory Medicine, University of Calgary and Calgary Laboratory Services, Calgary, AB Canada; 2 Departments of Oncology and Medicine, University of Calgary, Calgary, AB Canada; 3 Department of Pathology, University Kebangsaan Malaysia, Kuala Lumpur, Cheras Malaysia; 4 Division of Haematology and Transfusion Medicine, University of Calgary/Calgary Laboratory Services, Room 7522, 7th floor, McCaig tower building; 3134 Hospital drive NW, Calgary, AB T2N 5A1 Canada

**Keywords:** Acute leukemia, Chromosomal translocation, Fusion transcript, Fluorescent in-situ hybridization (FISH), Prognosis

## Abstract

**Background:**

The World Health Organization (WHO) classification system defines recurrent chromosomal translocations as the sole diagnostic and prognostic criteria for acute leukemia (AL). These fusion transcripts are pivotal in the pathogenesis of AL. Clinical laboratories universally employ conventional karyotype/FISH to detect these chromosomal translocations, which is complex, labour intensive and lacks multiplexing capacity. Hence, it is imperative to explore and evaluate some newer automated, cost-efficient multiplexed technologies to accommodate the expanding genetic landscape in AL.

**Methods:**

“nCounter® Leukemia fusion gene expression assay” by NanoString was employed to detect various fusion transcripts in a large set samples (*n* = 94) utilizing RNA from formalin fixed paraffin embedded (FFPE) diagnostic bone marrow biopsy specimens. This series included AL patients with various recurrent translocations (*n* = 49), normal karyotype (*n* = 19), or complex karyotype (*n* = 21), as well as normal bone marrow samples (*n* = 5). Fusion gene expression data were compared with results obtained by conventional karyotype and FISH technology to determine sensitivity/specificity, as well as positive /negative predictive values.

**Results:**

Junction probes for *PML/RARA*; *RUNX1-RUNX1T1*; *BCR/ABL1* showed 100 % sensitivity/specificity. A high degree of correlation was noted for *MLL/AF4* (85 sensitivity/100 specificity) and *TCF3-PBX1* (75 % sensitivity/100 % specificity) probes. *CBFB-MYH11* fusion probes showed moderate sensitivity (57 %) but high specificity (100 %). *ETV6/RUNX1* displayed discordance between fusion transcript assay and FISH results as well as rare non-specific binding in AL samples with normal or complex cytogenetics.

**Conclusions:**

Our study presents preliminary data with high correlation between fusion transcript detection by a throughput automated multiplexed platform, compared to conventional karyotype/FISH technique for detection of chromosomal translocations in AL patients. Our preliminary observations, mandates further vast validation studies to explore automated molecular platforms in diagnostic pathology.

**Electronic supplementary material:**

The online version of this article (doi:10.1186/s13000-016-0541-z) contains supplementary material, which is available to authorized users.

## Background

In acute myeloid leukemia (AML) and acute lymphoblastic leukemia (ALL), chromosomal abnormalities play a critical role in pathogenesis and prognosis [1]. Molecular studies of these genomic anomalies have identified specific genes implicated in the process of leukemogenesis [2]. Specific recurrent chromosomal translocations and their fusion transcripts define the current WHO classification system for acute leukemia (AL) [3]. These genetic aberrations in combination with morphology, immunophenotype and clinical features are used to diagnose distinct types of AL and thereby dictate therapeutic approaches [4]. Clinical laboratories, worldwide, utilize standard FISH technology with or without conventional karyotype to investigate these chromosomal translocations [5]. Unfortunately, FISH is a labour intensive and expensive procedure with the limited potential for “multiplexing”. These limitations impact diagnosis and clinical management. In a small number of AL sub-types, fusion transcripts can be amplified through reverse transcription, polymerase chain reaction (RT-PCR) methodology [6]. However, clinical application of RT-PCR is narrow due to variation in breakpoints resulting in several splice regions [7]. Given these limitations and the expanding new discoveries of additional recurrent chromosomal translocations, there is a need to explore newer automated technologies to “accommodate” new fusion transcript discoveries and make this significant clinical diagnostic testing cost-effective and time efficient.

Digital RNA quantification by NanoString Technologies is an automated, multiplexed platform, which can accurately measure up to 800 specific targets. “Leukemia Fusion Gene Expression Assay” by NanoString has a unique design of junction probes, which target the unique sequence spanning the fusion junction of the two exons. It has been claimed that a high degree of sensitivity and specificity has been achieved through sequence modification. However, no clinical validation data are available to determine the utility of this automated technology in routine clinical practice. The objective of this study was to evaluate the potential application and clinical utility of “Leukemia Fusion Gene Expression Assay” in the diagnostic sub-classification of acute leukemia patients. To this effect, we investigated fusion transcripts in a large cohort of AML and ALL patients utilizing genomic RNA from diagnostic bone marrow biopsies. We then correlated these results with data obtained by routine FISH and conventional karyotype. Our results provide unique preliminary data comparing a traditional platform with a novel automated technology, which provides potential promise for future adoption in the diagnosis and management of acute leukemia patients.

## Methods

University of Calgary ethics committee approved this study (REB-13-0380-MODI; dated August 21, 2013). The diagnosis was confirmed according to the WHO 2008 classification [3]. We employed adequate representation of samples with recurrent chromosomal translocation (true positive) in AML (*n* = 23) and ALL (*n* = 26) while AML with normal cytogenetics (*n* = 19), or complex cytogenetics (*n* = 21) and normal bone marrow (*n* = 5) samples were used as negative controls (true negative).

### Karyotype and fluorescence in-situ hybridization

Complete karyotype analysis at diagnosis was performed on all leukemic patients and followed a standardized laboratory protocol. Banded chromosomes (20 metaphases) were examined for each patient sample utilizing the G-banding technique. All patients (100 %) with diagnosis of ALL (*n* = 26) were subjected to additional FISH studies utilizing a panel of four probes (TCF3-PBX1; MLL-AF4; BCR-ABL and ETV6-RUNX1). Among AML patients, FISH studies were performed only as second tier test, as required by findings on karyotype analysis (8/23; 35 %). Dual color dual fusion or dual color break part FISH probes (Abbott, Markham ON) were employed to confirm translocations as dictated by clinical requirements or the karyogram. The results of these diagnostic tests performed on aspirate material were retrieved from patient records for comparison with digital quantification of fusion transcripts by NanoString technology (see below).

### Gene expression analysis

We employed nCounter® Leukemia Fusion Gene Expression Assay utilizing the NanoString platform (NanoString Technologies, Seattle, WA) to evaluate the expression of fusion transcript (Additional file 1: Table S1). RNA was isolated using the High Pure RNA Paraffin Kit (Roche, Basel, Switzerland) utilizing duplicate cores (2-mm), harvested off areas with maximum tumor cell population in the FFPE tissue block of diagnostic bone marrow specimen. All bone marrow biopsies were subjected to a standardized, short term (1 h 45 min) formic acid decalcification protocol. The RNA concentration was quantified using UV spectroscopy (Nanodrop Technologies, Wilmington, DE) and integrity assessed using a Bio-analyzer 2100 and RNA Nano Chip assay (Agilent Technologies, Wilmington, DE). The necessary probes and reagents were purchased from NanoString, and the nCounter analysis was performed according to the manufacturer’s instructions. Probes were hybridized to 800 ng of total RNA for 20 h at 65 °C. Hybridized reactions were purified using nCounter Prep Station and data collection was performed on nCounter Digital Analyzer. Raw counts were normalized using nSolver Analysis Software v3.0. Background subtraction was performed for each sample by subtracting the mean of 8 negative controls from all data points. Raw counts were further normalized to the six positive controls, included in each CodeSet, and to the two housekeeping genes TBP and GUSB.

### Sensitivity, specificity, positive and negative predictive values

The sensitivity, specificity, positive and negative predictive values were determined for the seven fusion transcripts by comparing signal-to-noise ratio (SNR). The median binding value in true negative samples was considered as background or “noise”. Minimum of ten-fold or more difference between SNR was considered as the positive result and was used for further calculations.

### Statistical analysis

We used nSolver software v3.0 (NanoString Technologies) for the normalization of raw counts. Heat map and principle component analyses were performed on Qlucore Omics Explorer v3.2 (Lund, Sweden). Statistical analysis was performed using SPSS software v21.0 (IBM, Armonk, NY). All computed results having two-sided *P* value < 0.05 were considered significant.

## Results

**Fig. 1:**
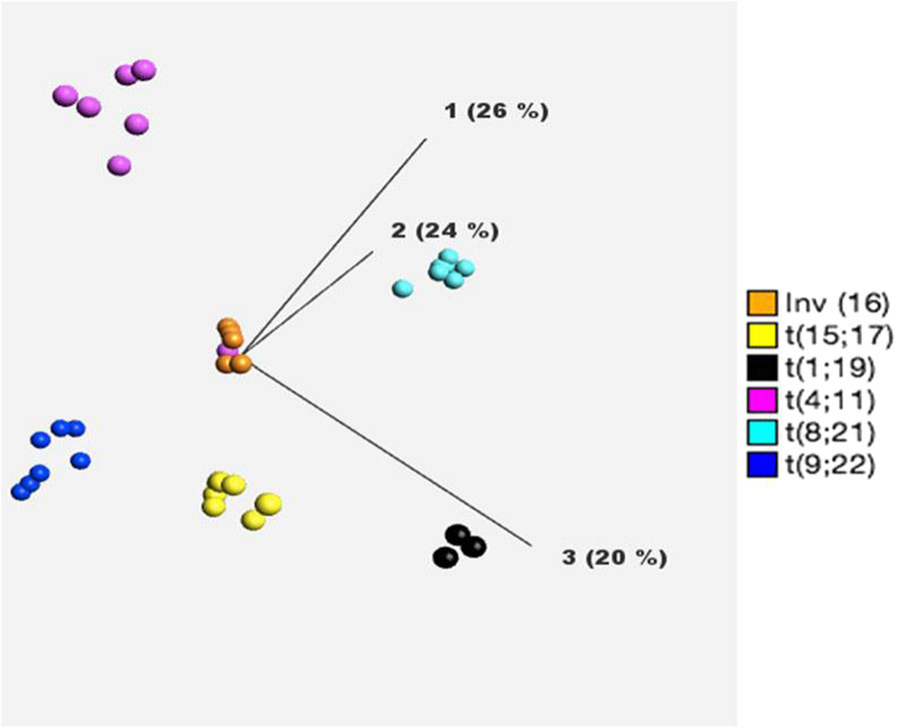
Principal component analysis (PCA) plot of AL samples with recurrent translocations [except t (12;21)]; showing discrete clustering of samples as detected by digital quantification of fusion transcripts, utilizing NanoString technology

A total of 94 (63 AML, 26 ALL and five control bone marrow) samples were analysed. AML patients included 42 men and 21 women (M: F 2:1) with a median age of 63 years (range 5–87 years). Twenty-six ALL patients comprised of 17 men and 09 women (M: F 1.8:1) with a median age of 12 years (range 2–63 years) in this cohort. Table 1 outlines the sensitivity, specificities, and positive and negative predictive values for each transcript. Figure 1 outlines, principal component analysis (PCA) plot based on digital quantification of various fusion transcripts and their correlation with chromosomal/FISH results. In AML category, digital quantification for *PML/RARA* and *RUNX1-RUNX1T1* correlated 100 % with t(15;17) and t(8;21) respectively; while non-specific positivity was not seen in any other samples across entire cohort. *CBFB-MYH11* fusion probes showed “false positivity” in AML samples with complex and normal karyotype. In 4/8 (50 %) of AML samples with inv(16) or t(16;16); false negative reaction was noted. In ALL group, *BCR-ABL1* fusion probes performed seamlessly with 100 % sensitivity and specificity. *TCF3-PBX1* and *MLL-AF4* fusion probes showed 100 % specificity but sensitivity ranged up to 86 %. Probes for *ETV6/RUNX1* fusion transcript performed poorly and showed only 50 % sensitivity but 98 % specificity.

**Table 1:** Specificity/sensitivity/positive predictive value and negative predictive value for each transcript as detected by digital RNA quantification

	No (n)	Result (Pos./ Neg.)	Sensitivity (%)	Specificity (%)	PPV^a^ (%)	NPV^b^ (%)
Acute Myeloid Leukemia (AML)						
AML1-ETO	8	8/0	100	100	100	100
PML-RARA	8	8/0	100	100	100	100
CBFB-MYH11	7	4/3	57	100	100	97
Acute Lymphoblastic Leukemia (ALL)						
TCF3-PBX1	4	3/1	75	100	100	99
MLL-AF4	7	6/1	85	100	100	99
BCR-ABL	7	7/0	100	100	100	100
ETV6-RUNX1	8	1/7	12	92	50	92
AML - Normal Karyotype	19	0/19	N/A	100	N/A	100
AML - Complex Karyotype	21	1/20	N/A	99	N/A	100
Normal Bone Marrow	5	0/5	N/A	100	N/A	100

^a^ *PPV* positive predictive value; ^b^ *NPV* negative predictive value

## Discussion

In WHO classification for AL, chromosomal translocations are central to diagnosis, sub classification and prognosis. Conventional cytogenetics, followed by FISH in specific cases, is the gold standard to detect these chromosomal aberrations, but poses the challenge regarding subjectivity, manual labour, cost and turnaround time. There is a need to explore automated, multiplexed, throughput and cost-efficient technologies to serve rapidly expanding discoveries of novel fusion transcripts in haematological malignancies. A large cohort of AL samples were analysed by multiplexed, automated digital quantification in parallel with conventional karyotype/FISH technology. We observed a high degree of correlation (>90 % sensitivity and specificity) for different translocations that are frequent in clinical practice. The complete correlation was noted for t(8;21) between conventional karyotype/FISH and digital fusion RNA quantification results. This high degree of correlation can be related to the generation of only two fusion transcripts by this translocation [8]. t(8;21) can also involve several chromosomes, and these variant rearrangements can be cryptic and easily overlooked by conventional G-banding technique [9]. However, it was interesting to note that none of these cryptic fusion transcripts were detected in a large cohort of AL patients with normal and complex cytogenetics. Similarly, almost complete correlation was also noted for t(15;17); t(9;22) and t(4;11) translocations. Hence, we established the potential for clinical utility of this automated throughput platform of digital quantification as a possible alternate to current and widely used FISH technique.

Multiplexed molecular techniques present opportunities in procedural efficiencies, but also pose challenges regarding cross-reactivity and false positivity [10, 11]. Our extensive series of AL sample sets with normal and complex cytogenetic provided an adequate safety net to detect any cross-reactivity of fusion probes. We noted minimal to no false positive reactions in the majority of fusion probes. These results are encouraging in our small patient sample; however, a more robust validation is required using a larger cohort. In AL patients with t(12;21) (p13;q22), we noted discordance between FISH results and *ETV6-RUNX1* fusion probe. The breakpoints in t(12;21) (p13;q22) are variable within the several thousand base pairs of ETV6 as well as RUNX1 introns. In spite of micro-clustering of breakpoints in ETV6 and RUNX1 introns, it is still difficult to identify or to sequence the breakpoints by even streamlined long‐distance PCR in t(12;21) [12]. Hence, it appears that fusion transcript *ETV6-RUNX1* is beyond the scope of this assay at this stage. We also noted that inv(16) showed the most variable pattern and weak correlation with FISH results. This translocation results in more than ten differently sized *CBFB-MYH11* fusion transcripts, which may explain variable sensitivity [13]. The probes against *CBFB-MYH11* showed cross-reactivity with few samples harbouring normal or complex karyotype. Since there is marked heterogeneity of breakpoints in MYH11 gene, false negative rates could be high [14]. False positivity in inv 16 cases has also been reported by RT-PCR [15]. These observations mandate re-designing of some probes for this assay for enhanced sensitivity.

## Conclusion

Our study demonstrates initial evaluation for fusion transcript detection by a throughput automated multiplexed platform for AL patients as an alternate solution to current complex, manual and labour intensive karyotyping /FISH techniques. Our preliminary study requires validations by future studies to evaluate these emerging technologies for their potential adoption in clinical laboratories. These expression assays offer significant capacity for expansion to accommodate additional future fusion transcripts discoveries of diagnostic or prognostic value in AL patients. It also has an immense ability to include gene expression signature as a diagnostic or prognostic tool for AL patients.

## Additional file

Additional file 1: Table S1. Raw data. (XLS 67 kb)

## Abbreviation

WHO: World Health Organization
AL: Acute leukemia
FISH: Fluorescent in-situ hybridization
FFPE: Formalin fixed paraffin embedded
AML: Acute myeloid leukemia
ALL: Acute lymphoblastic leukemia
RT-PCR: Reverse transcription-polymerase chain reaction
SNR: Signal-to-noise ratio
SPSS: Statistical package for the social sciences
PCA: Principal component analysis
